# Multi-scale guided feature extraction and classification algorithm for hyperspectral images

**DOI:** 10.1038/s41598-021-97636-2

**Published:** 2021-09-15

**Authors:** Shiqi Huang, Ying Lu, Wenqing Wang, Ke Sun

**Affiliations:** 1 Xi’an University of Posts and Telecommunications, Xi’an, 710121 China; 2 Xi’an Key Laboratory of Advanced Control and Intelligent Processing, Xi’an, 710121 China

**Keywords:** Applied optics, Imaging and sensing

## Abstract

To solve the problem that the traditional hyperspectral image classification method cannot effectively distinguish the boundary of objects with a single scale feature, which leads to low classification accuracy, this paper introduces the idea of guided filtering into hyperspectral image classification, and then proposes a multi-scale guided feature extraction and classification (MGFEC) algorithm for hyperspectral images. Firstly, the principal component analysis theory is used to reduce the dimension of hyperspectral image data. Then, guided filtering algorithm is used to achieve multi-scale spatial structure extraction of hyperspectral image by setting different sizes of filtering windows, so as to retain more edge details. Finally, the extracted multi-scale features are input into the support vector machine classifier for classification. Several practical hyperspectral image datasets were used to verify the experiment, and compared with other spectral feature extraction algorithms. The experimental results show that the multi-scale features extracted by the MGFEC algorithm proposed in this paper are more accurate than those extracted by only using spectral information, which leads to the improvement of the final classification accuracy. This fully shows that the proposed method is not only effective, but also suitable for processing different hyperspectral image data.

## Introduction

With the rapid development of aerospace technology, the spectral resolution of remote sensing imaging technology has been greatly improved. Hyperspectral remote sensing is a kind of continuous imaging technology for ground target information using imaging spectrometer. Compared with other remote sensing images, hyperspectral images can more accurately reflect the state of the target in the image space and the characteristics in the spectral space. Moreover, the spectral data can be analyzed and processed more reasonably and effectively. Therefore, hyperspectral imaging technology has more advantages in ground object recognition and classification, and it has been widely used in many fields^1–8^. However, hyperspectral images also have many bands with strong correlation and high data redundancy, and feature extraction is one of the main methods to effectively solve these problems.

The purpose of feature extraction is to use different methods to select bands or features that contain large amount of information to reduce the redundancy of data. The common spectral feature extraction algorithms include principal component analysis (PCA), independent component analysis (ICA) and linear discriminant analysis (LDA)^9^. These dimensionality reduction methods all extract spectral features of hyperspectral images through linear transformation. Among them, PCA is the most commonly used linear feature extraction method. In this method, the input data is transformed by using the transformation matrix, and the feature with the largest contribution of the corresponding variance in the data is retained, and then the data dimension is reduced. On this basis, a series of PCA improved algorithms were proposed^10–15^. For example, PCA algorithm was applied to hyperspectral image, and the method of selective principal component analysis was proposed^10^. This method has very good prediction results for pixels with small number of features. According to the high correlation of hyperspectral images, a more advanced segment using PCA dimension reduction method was further proposed^11^. The morphological principal component analysis was proposed and applied to hyperspectral image feature extraction^14^. Similarly, ICA theory is considered as an extension of PCA, which is based on the assumption that data are independent of each other, and is also used to extract features and make classification for hyperspectral images^16–19^. For example, the^19^ used PCA and ICA to obtain PCA features and edge features of hyperspectral remote sensing images, and then used deep convolution neural network to realize the fusion and classification of spatial-spectral features.

The above method only considers the spectral information of the image and ignores the existence of spatial features when extracting features. Image spatial features mainly include regional shape features, texture features and morphological filtering features. Since hyperspectral images have the characteristics of space-spectrum integration, if the spatial information and spectral information of hyperspectral images are used to extract their features at the same time, the effect will be better. At present, the method of feature extraction combining spatial and spectral information of hyperspectral image is a hot field in hyperspectral remote sensing^20–23^. The commonly used spatial feature extraction methods include discrete wavelet transform, Gabor filtering, sparse representation and local binary pattern. The local binary pattern (LBP) is a texture feature extraction method, and it is also widely used in spatial-spectral feature extraction of hyperspectral images^24^. A hyperspectral image classification method based on the combination of dual channel CNN and LBP was proposed, and good classification results were obtained^24^. The hyperspectral image classification algorithm based on super-pixel and multi-core sparse representation was proposed, and achieved the expected classification effect^25^.

The spatial features extracted in the above research are generally single scale features. Because it is very difficult for using single scale feature to accurately express the differences between object categories, and cannot well distinguish the boundary of objects, the idea of multi-scale feature extraction has been widely used in the field of hyperspectral image processing^26–29^. The core idea of multi-scale feature extraction is to realize the abstraction of image information at different scales. Among them, the control of scale parameters has the following situations, such as the designed filter banks, different sizes of structural elements and different sizes of processing windows. For example, morphological filtering, which used circular structural elements of different sizes to perform open and close operations on images, could better smooth noise^30^. However, the traditional morphological filtering cannot effectively keep the information of the edge structure of the ground object while realizing the smooth of the ground object. The guided filtering algorithm not only has better filtering ability, but also has better edge preserving ability^31^, so it has been widely used in image fusion, image enhancement and image feature extraction^32–37^. For example, a very high-resolution remote sensing image classification method based on guided filtering multi-scale super-pixel features was proposed in literature^37^, which could obtain multi-scale feature information by setting different filter window sizes, so as to improve the classification accuracy and calculation efficiency of detail information. On this basis, this paper introduced the idea of guided filtering into hyperspectral image feature extraction, and proposed a multi-scale guided feature extraction and classification (MGFEC) algorithm for hyperspectral images. Several hyperspectral remote sensing image datasets were used to verify the experiment, and good classification results were obtained.

The current paper makes several contributions to the field. The first is the conceptual design for multi-scale guided filtering to get features; the second is that random blocks are extracted as convolution kernel, and features are further extracted for the deep level feature.

## Guided filtering

Guided filter (GF) is a very efficient edge-preserving filter with better performance than bilateral filter. Through local linear model, the information of guiding image is skillfully added to the input image, so that the output image can obtain some enhanced features, such as edge preservation and enhancement. Its core technology is a linear shifting filtering equation^31,38^. Guided filtering algorithm guides the input image to complete the filtering process. In the process of realization, it is considered that there is a linear processing relationship between the pixel points of the guided image and the output image, and there is a spatial filtering relationship between the input image and the output image. Therefore, the final output image is similar to the input image in structure, and similar to the guided image in texture details.

Usually, it can be considered that an image is composed of background layer and foreground layer (target or detail). Therefore, the filtering and enhancement of the image are almost to suppress the background layer information and to enhance the detail layer information. In most cases, it is a compromise between them. Similarly, the guided filtering process also follows this thinking. Assume that $I$ is the input image to be filtered, $G$ is the guided image, and $F$ is the filtered image to be output. According to the idea of image guided filtering, there is a linear relationship between the guided image $G$ and the output image $F$ in the local filtering window $W_{k}$, and the relationship model can be expressed by Eq. (1).

1 $$ F_{i} = a_{k} G_{i} + b_{k} { ; }\quad \forall i \in W_{k} $$

It can be seen in Eq. (1) that as long as the values of the linear coefficients $a_{k}$ and $b_{k}$ are calculated, and then the pixel value $F_{i} (m,n)$ of the filtered image can be obtained through the pixel value $G_{i} (m,n)$ of the guide image.

In the filtering window $W_{k}$, to minimize the difference between the input image $I$ and the output image $F$, the cost function $E( \cdot )$ can be constructed to achieve this. Its mathematical model is shown in Eq. (2).

2 $$ E(a_{k} ,b_{k} ) = \sum\limits_{{i \in W_{k} }} {\left[ {(F_{i} - I_{i} )^{2} + \varepsilon a_{k}^{2} } \right]} = \sum\limits_{{i \in W_{k} }} {\left[ {(a_{k} G_{i} + b_{k} - I_{i} )^{2} + \varepsilon a_{k}^{2} } \right]} $$

where $\varepsilon$ is the regularization parameter greater than zero, which is to prevent the coefficient $a_{k}$ from being too large, and it is conducive to maintaining the overall stability of the data.

The coefficients $a_{k}$ and $b_{k}$ in Eqs. (1) and (2) can be solved by the least square method, and its calculation equation is shown in Eq. (3).

3 $$ \left\{ \begin{gathered} a_{k} = \frac{{\frac{1}{|\omega |}\sum\limits_{{i \in W_{k} }} {G_{i} I_{i} - \mu_{k} \overline{I}_{k} } }}{{\sigma_{k}^{{2}} + \varepsilon }} \hfill \\ b_{k} = \overline{I}_{k} - a_{k} \mu_{k} \hfill \\ \end{gathered} \right. $$

where $\mu_{k}$ and $\sigma_{{\text{k}}}^{2}$ respectively represent the mean value and variance of all pixels in the local filtering window $W_{k}$ of the guided image $G$. $|\omega |$ is the number of pixels in the window $W_{k}$, and its size is $r \times r$. $\overline{I}_{k}$ is the mean value of all pixels of the local window with the same size of the window $W_{k}$ in the input image $I$.

During processing, as the window $W_{k}$ moves, the pixel $i$ will be contained in multiple different windows $W_{k}$ that overlay it. Because the pixels and their values in different windows are different, the values of coefficients $a_{k}$ and $b_{k}$ in each window calculated by Eq. (3) are also different. To make the value of $a_{k}$ and $b_{k}$ more accurate, it is necessary to sum and average the corresponding values obtained in all windows $W_{k}$ containing pixel $i$. Therefore, the final expression of the output image $F_{i}$ can be obtained by transforming Eq. (1), and it is given by

4 $$ F_{i} = \overline{a}_{k} G_{i} + \overline{b}_{k} { ; }\forall i \in W_{k} $$

where the average coefficient $\overline{a}_{k}$ and $\overline{b}_{k}$ can be calculated by Eq. (5).

5 $$ \left\{ {\begin{array}{*{20}l} {\overline{a}_{k} = \frac{1}{|\omega |}\sum\limits_{{k \in W_{k} }} {a_{k} } } \hfill \\ {\overline{b}_{k} = \frac{1}{|\omega |}\sum\limits_{{k \in W_{k} }} {b_{k} } } \hfill \\ \end{array} } \right. $$

In the process of guided filtering, the size of window $r$ and the regularization parameter $\varepsilon$ are the two important parameters, and their different values will affect the final filtering results. The larger the filtering window is, the more obvious the smoothing effect is. The smaller the filtering window, the more details are preserved. The larger the regularization parameter is, the stronger the regularization ability is, but the influence on the filtering effect is limited.

## Description of MGFEC algorithm

**Figure 1:**
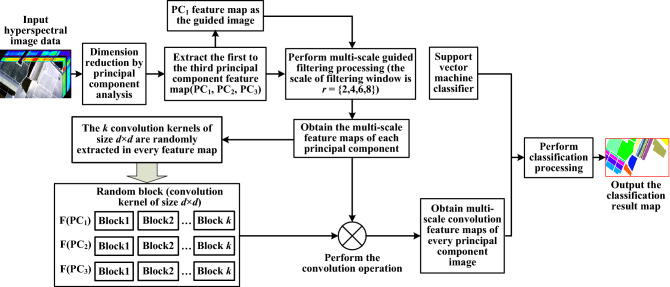
Schematic diagram of MGFEC algorithm.

Hyperspectral remote sensing image data cube is characterized by many bands and high correlation between bands, so it usually need dimension reduction. In the MGFEC algorithm proposed in this paper, firstly, PCA is used to process hyperspectral image data to reduce its dimension and facilitate subsequent feature extraction and processing. Then, the first three principal component images are acquired and used as input images at the same time, while the first principal component image is used as the guided image. By setting different filter window sizes to complete the guided filtering processing, the multi-scale feature map of each principal component is obtained. In each feature map, $k$ blocks are randomly extracted as convolution kernel template, and then convolution operation is performed with each feature map to further extract deep multi-scale features. Finally, the classification of hyperspectral remote sensing image is realized by using the classifier to classify the feature image. The principle block diagram of MGFEC algorithm is shown in Fig. 1, and the main implementation steps are as follows.

1. Input hyperspectral image data. The original hyperspectral data is denoted with $X$, and $X \in {\mathbb{R}}^{m \times n \times L}$, $m \times n$ is the size of each band image and $L$ represents the number of bands.
2. Reduce the dimension of hyperspectral remote sensing image data. The principal component analysis (PCA) theory is used to process the hyperspectral image data, and each principal component feature map is obtained. Because the first three principal components contain more than 95% information, they are extracted for subsequent processing. The PCA algorithm is to transform a set of possible correlation variables into a group of linear unrelated variables through orthogonal transformation. The converted variables are called the main components. The specific calculation steps of PCA algorithm are as follows.
  - Let the original hyperspectral image data be represented by matrix ${\mathbf{X}}$, which is shown in Eq. (6). Each band image is expanded into a row vector as a row of matrix ${\mathbf{X}}$
    6 $$ {\mathbf{X}}{ = }\left\{ {\begin{array}{*{20}c} {x_{11} } & \cdots & {x_{1N} } \\ \vdots & \vdots & \vdots \\ {x_{L1} } & \cdots & {x_{LN} } \\ \end{array} } \right\} $$
    where $L$ is the number of bands, $N$ is the size of the image, i.e. $N = m \times n$.
  - The hyperspectral image data is standardized to get matrix ${\mathbf{A}}$, and the calculation formula is as follows.
    7 $$ {\mathbf{A}}{ = }\left\{ {\begin{array}{*{20}c} {a_{11} } & \cdots & {a_{1N} } \\ \vdots & \vdots & \vdots \\ {a_{L1} } & \cdots & {a_{LN} } \\ \end{array} } \right\} $$
    where $a_{ij} = x_{ij} - \mu_{i}$, $i = 1,2, \ldots ,L$, $j = 1,2, \ldots ,N$. $\mu_{i} = (\sum\nolimits_{j = 1}^{N} {x_{ij} } )/N$, it is the mean of the $i$ th line in the original hyperspectral data ${\mathbf{X}}$
  - The covariance matrix ${\mathbf{R}}$ of standardized matrix ${\mathbf{A}}$ is calculated as follows.
    8 $$ \left\{ {\begin{array}{*{20}l} {{\mathbf{R}}{ = }\left\{ {\begin{array}{*{20}c} {r_{11} } & \cdots & {r_{1N} } \\ \vdots & \vdots & \vdots \\ {r_{L1} } & \cdots & {r_{LN} } \\ \end{array} } \right\}} \hfill \\ {r_{ik} = \frac{{\sum\limits_{i = 1}^{L} {(a_{ij} - \overline{{a_{i} }} )(a_{ik} - \overline{{a_{k} }} )} }}{{\sqrt {\sum\limits_{i = 1}^{L} {(a_{ij} - \overline{{a_{i} }} )^{2} (a_{ik} - \overline{{a_{k} }} )^{2} } } }}} \hfill \\ \end{array} } \right. $$
    where $$j,k = 1,2, \ldots ,N$$, $$a_{i} = \frac{1}{L}\sum\limits_{i = 1}^{L} {a_{ij} }$$.
  - The eigenvalues of covariance matrix ${\mathbf{R}}$ and corresponding eigenvectors are calculated. Using the characteristic equation $\left| {R - \lambda I_{L} } \right| = 0$, we can get $L$ eigenvalues of the covariance matrix ${\mathbf{R}}$, which are arranged from big to small, namely $\lambda_{1} \ge \lambda_{2} \ge \cdots \ge \lambda_{L} \ge 0$, and the corresponding eigenvector is $v_{1} ,v_{2} , \cdots ,v_{L}$.
  - The eigenvectors corresponding to the first three eigenvalues constitute matrix ${\mathbf{V}}$, and the calculation formula is as follows.
    9 $$ {\mathbf{V}}{ = }\left\{ {\begin{array}{*{20}c} {v_{11} } & {v_{12} } & {v_{13} } \\ \vdots & \vdots & \vdots \\ {v_{N1} } & {v_{N2} } & {v_{N3} } \\ \end{array} } \right\} $$
  - Output the data after dimensionality reduction, $PC = V^{T} \cdot X$, and the calculation formula is as follows.
    10 $$ PC{ = }\left\{ {\begin{array}{*{20}c} {PC_{11} } & \cdots & {PC_{1N} } \\ {PC_{21} } & \vdots & {PC_{2N} } \\ {PC_{31} } & \cdots & {PC_{3N} } \\ \end{array} } \right\}{ = }\left\{ {PC_{1} ,PC_{2} ,PC_{3} } \right\}^{T} $$
    where $PC_{1}$, $PC_{2}$ and $PC_{3}$ are the first, the second and the third principal component, respectively.
    Due to the strong correlation between adjacent bands in hyperspectral image data, it is necessary to whiten each principal component separately, so that the variance between different bands is similar, so as to reduce the redundancy between different bands, which is conducive to the subsequent feature extraction. The whitening process here refers to the whitening of PCA, namely, to normalize the variance of each one-dimensional feature of the data after PCA dimensionality reduction. In fact, it is to divide the data on each feature axis by the corresponding eigenvalue. Because the eigenvalue is equal to the variance of the dimension corresponding to the data in the rotated coordinates, the normalization amplitude on each feature axis is achieved. The specific calculation formula is as follows.
    11 $$ PC_{white,w} = \frac{{PC_{w} }}{{\sqrt {\lambda_{w} } }}; \, w \in [1,2,3] $$
    where $\lambda_{w}$ is the eigenvalue corresponding to the $w$th dimension eigenvector in the matrix $PC$ obtained after principal component transformation.
3. Obtain multi-scale guided filtering feature maps. The first three principal components are respectively guided and filtered. Firstly, it needs to build a guided image. The first principal component $PC_{1}$ in step (2) is taken as the guided image, and the first to the third principal components $PC_{1} \sim PC_{3}$ are as the input image. Secondly, it needs to set the size of filter window. Here the filter window is set to four different scales, i.e. 2, 4, 6 and 8. Thirdly, different scale guided filtering is applied to different principal component feature maps. The result of guided filtering is that each principal component image can obtain four feature maps of different scales, and then stack all the feature maps of each principal component to form a feature set of multi-scale guided filtering. The calculation formula is as follows.
  12 $$ F(PC_{i} ) = \{ f_{G}^{1} (PC_{i} ),f_{G}^{2} (PC_{i} ), \ldots ,f_{G}^{r} (PC_{i} )\} ; \, r \in [2,4,6,8] $$
  where $f_{G}^{r} (PC_{i} )$ represents the multi-scale filtering feature obtained by the guided image $G$ to the $i$th principal component $PC_{i}$ when the filtering window scale is $r$
4. Acquire random patches. After multi-scale guided filter feature extraction, 12 multi-scale feature maps were obtained from the first three principal component images. Then, $k$ pixels are randomly selected in each scale feature map, and around each pixel, the image patch with window size $d \times d$ is selected to obtain $k$ image blocks. Here $d$ is the size of the random patch.
5. The convolution operation is performed and the feature map is obtained. The $k$ random blocks obtained in step (4) are taken as convolution kernels, and they are convolved with the feature set $F$, respectively. Finally, we can get $k \times 12$ feature maps, stack them and build the feature set $F \in {\mathbb{R}}^{m \times n \times 12 \times k}$, which will be classified in step (6).
6. Set up classifiers and carry out classification processing. Support vector machine (SVM) is a very good classifier, especially for small sample data sets. In terms of classification accuracy, SVM is better than most classifiers, so it has been widely used. Therefore, in MGFEC algorithm, the efficient SVM classifier is chosen to classify the acquired feature sets. The classification process is as follows.
  - Data sets are divided into different subsets. From the multi-scale feature set, 75% of the feature maps are randomly selected as the training sample subset, and the remaining features are selected as the test sample subset.
  - The training data set is used to train the model, then the trained model is used to predict and classify the whole test data, and finally the classification results are obtained.

## Discussion and analysis of experimental results

### Experimental data

**Figure 2:**
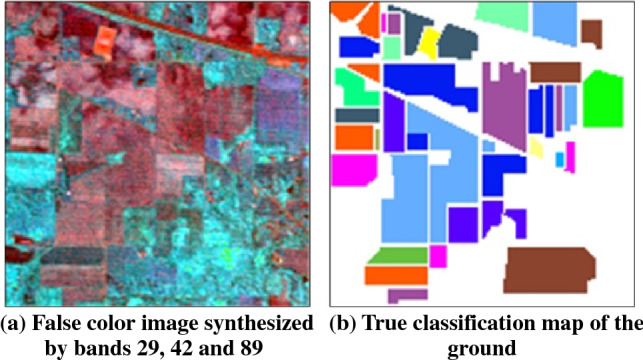
The image of Indian pines area and real classification map of ground.

To verify the effectiveness of the MGFEC algorithm proposed in this paper, three groups of hyperspectral data and different feature extraction methods are compared. The basic information of hyperspectral image data used in the experiment is shown in Table 1. These data come from three different ground scene areas. Among them, Indian pine data was obtained by AVIRIS sensor. The size of image data is 145 × 145, and the spectral imaging wavelength range is 0.4–2.5 nm. There are 224 bands, including 200 effective bands. Figure 2a is a false color image composed of bands 29, 42 and 89, and Fig. 2b is the real ground situation of the data, with a total of 16 crop categories, as shown in Table 2.

**Table 1:** Basic information of experimental hyperspectral data.

Sensor	Scene	Band number	Size	Scene features
AVIRIS	Indian Pines	200	145 × 145	Farmland
ROSIS	Pavia University	103	610 × 340	Suburbs, buildings
AVIRIS	Salinas Valley	204	512 × 217	Farmland

**Table 2:**
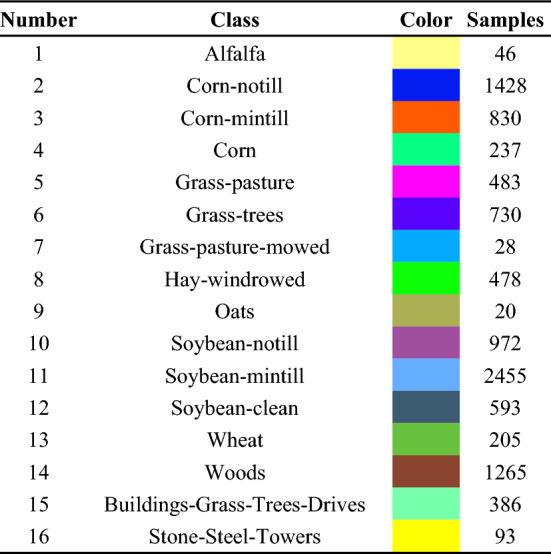
Information of ground object types in Indian pines area.

**Figure 3:**
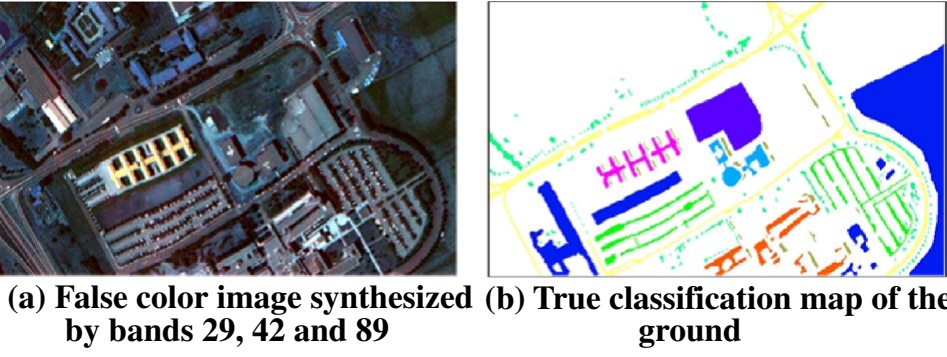
The image and ground truth classification map of Pavia university area.

The data of Pavia university area was obtained by ROSIS sensor, with spatial resolution of 1.3 m and image size of 610×340. The sensor has a total of 115 bands. After processing, Pavia university data has 103 effective bands. The image contains nine different types of ground objects. The data is shown in Fig. 3, in which Fig. 3a is a false color image composed of wavelengths 29, 42 and 89, and Fig. 3b is the corresponding real ground classification map. The types of ground objects are shown in Table 3.

**Table 3:**
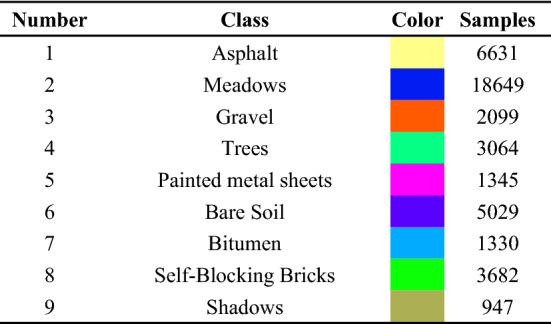
The classification information of ground objects in Pavia university area.

**Figure 4:**
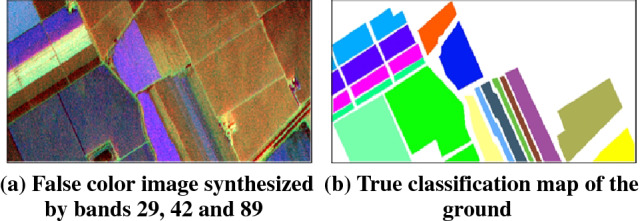
The image and ground truth classification map of Salinas Valley area.

Salinas Valley data was also captured by AVIRIS sensors in Salinas Valley, California. The spatial resolution of the data is 3.7 m, and the size of the image is 512×217. There are 224 bands in the original data. After removing the bands with serious water vapor absorption, there are 204 effective bands left. The data imaging area contains 16 crop categories, and the specific classification is shown in Table 4. Figure 4 shows the hyperspectral data, in which Fig. 4a is a false color image composed of 29, 42 and 89 bands, and Fig. 4b is a true situation of ground type.

**Table 4:**
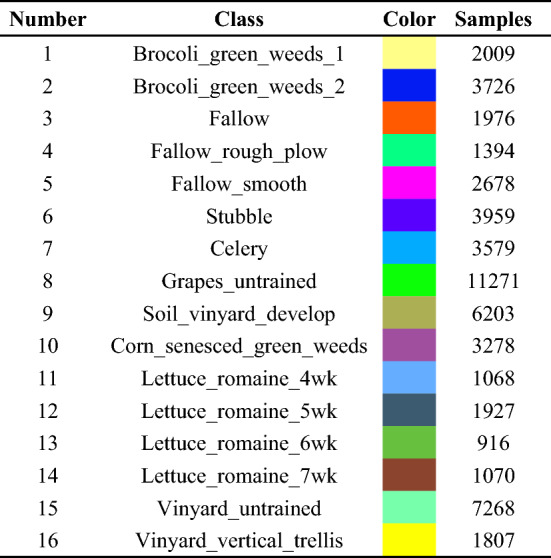
The classification information of ground objects in Salinas Valley area.

### Comparative experiments of different methods

**Figure 5:**
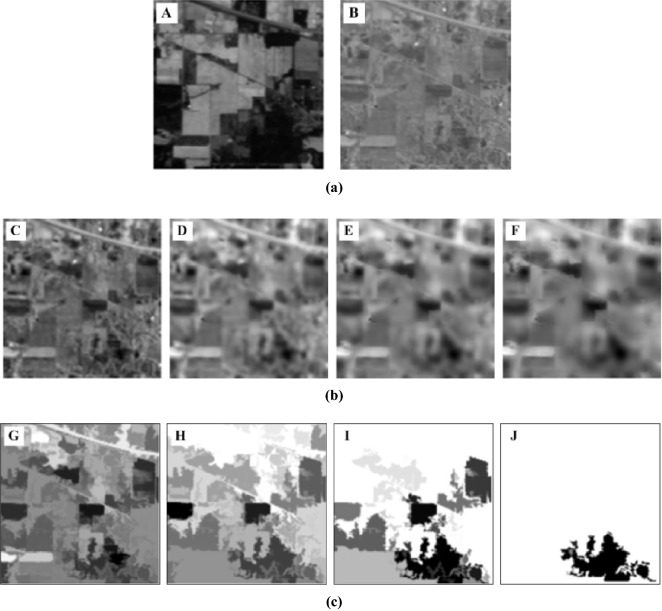
Results of multi-scale features extracted by different methods. (**a**) Dimension reduction results of PCA theory of Indian pines data (A. the first principal component image, B. the second principal component image). (**b**) Multi scale features extracted by MGFEC algorithm (C-F denotes feature maps with scales of 2, 4, 6 and 8 respectively). (**c**) Multi scale features extracted by EMP algorithm (G-J denotes feature maps with scales of 2, 4, 6 and 8 respectively).

**Figure 6:**
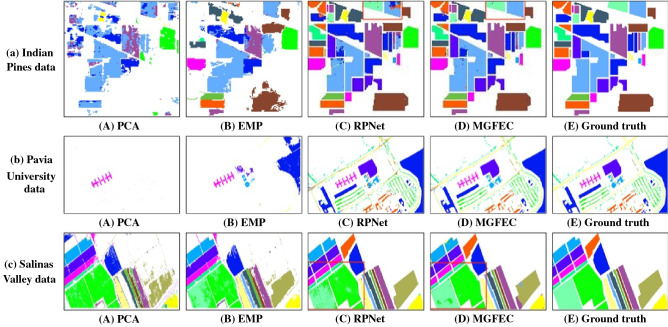
Experimental results of different methods and different data.

**Figure 7:**
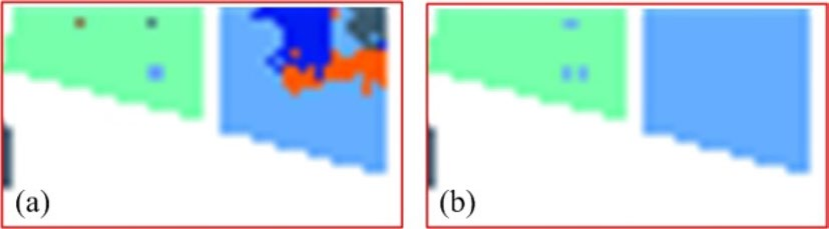
Comparison of the amplification effect of red box area in Fig. 6a.

**Figure 8:**
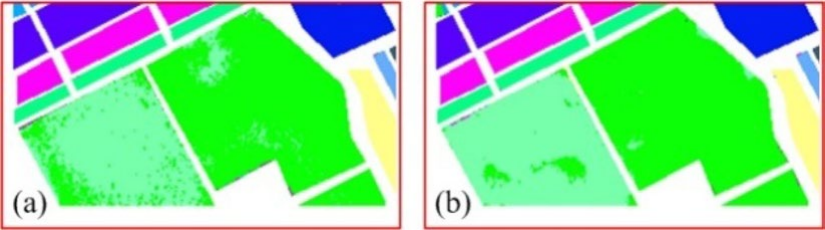
Comparison of the amplification effect of red box area in Fig. 6c.

In addition to the MGFEC algorithm proposed in this paper, there are principal component analysis (PCA), extended morphological profile (EMP)^39^ and random patch network (RPNet)^40^ in the comparative experiment. In the experiment, PCA theory is used to reduce the dimension of hyperspectral remote sensing image data. Because the first three principal components contain more than 95% information of the original data, the first three principal components are used as the basic image for extracting spectral features, which are used for spatial feature extraction in the subsequent methods. The above methods and experimental data were used to carry out comparative experiments, and the obtained experimental results were shown in Figs. 5–8. In these experiments, the parameters were set as follows.

1. MGFEC algorithm. For the guided filter, there are two key parameters to be set, namely, the size $r$ of the filter window and the regularization parameter $\varepsilon$. In MGFEC algorithm, the regularization parameter is set to 10^–4^, i.e. $\varepsilon = 10^{ - 4}$, and the spatial structure information of different scales of hyperspectral data is extracted by controlling the size $r$ of filter window. In order to make the results comparable, the size of the guided filter window is set to 2, 4, 6 and 8. In addition, when the algorithm principle and implementation steps are introduced in Sect. 3, the number of pixels $k$ selected in step (4) is set to 20, that is, the number of randomly extracted image blocks, and the size $d$ of each random blocks is set to 21.
2. EMP algorithm. The EMP algorithm uses different sizes of structural elements to complete the opening and closing operations of the original image, so as to achieve multi-scale structural feature extraction. In order to facilitate comparative analysis, the size of structural elements is set to 2, 4, 6 and 8, and each structural element is used for morphological filtering of each principal component image.
3. RPNet algorithm. The RPNet method based on deep learning directly extracts random blocks from the image as convolution kernel. In this process, no training is needed, and multi-scale convolution features can be obtained by convolution between the original image and different scale convolution kernel, which is the advantage of multi-scale of RPNet algorithm. In this paper, the number $k$ of convolution kernels is set to be 20 and the size $d$ of convolution window is 21.

Figure 5 shows a comparative experiment of multi-scale features using hyperspectral data from Indian pines. The first and the second principal component images obtained by PCA theory are shown in Fig. 5a. Figure 5A is the PC_1_ feature map and Fig. 5B is the PC_2_ feature map. MGFEC algorithm and EMP algorithm are used to extract multi-scale features from the second principal component feature map PC_2_. The first principal component image PC_1_ is only used as a guided image when MGFEC algorithm extracts features. The multi-scale features obtained by MGFEC algorithm and EMP algorithm are shown in Fig. 5b and Fig. 5c, respectively. In Fig. 5b, C-F represents the multi-scale features obtained with window sizes of 2, 4, 6 and 8, respectively. Similarly, in Fig. 5c, G-J represents the different features which are obtained with window sizes of 2, 4, 6 and 8, respectively.

It can be seen in Fig. 5 that when the window size is 2 and 4, the two methods can basically extract the structural features of ground objects. However, when the window size is 6 and 8, their extraction effect is relatively poor. At this time, MGFEC algorithm is better than EMP algorithm, and it can also better retain the main edge information of the image. At the same time, it is also known that with the increase of scale, the effect of feature acquisition by them is getting worse and worse. Through the comparative experiment of feature extraction, the conclusion can be gotten that the guided filtering method has better ability to retain the edge information of ground objects than the morphological method. Therefore, in MGFEC algorithm, the principle of guided filtering is used to extract multi-scale features.

The images shown in Fig. 6 are the results of classification of hyperspectral images of the above three regions by different methods. Figure 6a-c respectively represent the hyperspectral image data of different regions, corresponding to the data shown in Figs. 2–4 in turn. The results shown in Figs. 6A-D are the experimental results obtained by PCA, EMP, RPNet and MGFEC algorithms for classification of hyperspectral remote sensing image data in the same area, respectively. Figure 6E is the real classification map of the ground truth.

It can be seen in Fig. 6a, in the Indian pines scene, the PCA method only uses spectral features, so the classification result is not accurate, and salt and pepper phenomenon appears (as shown in Fig. 6A). This shows that only using spectral features cannot be a good way to classify ground objects. Therefore, researchers have proposed a series of space spectral feature joint classification algorithms for hyperspectral images, which have improved the salt and pepper phenomenon in the classification results to a certain extent. The EMP and RPNet methods combine the spatial features of the image, and introduce the multi-scale feature space, so the classification results are significantly improved, and the salt and pepper phenomenon is reduced, as shown in Fig. 6B,C. MGFEC algorithm not only obtains good classification results, but also has almost no salt and pepper phenomenon, which is shown in Fig. 6D. The reason is that MGFEC algorithm introduces the principle of guided filtering to extract features, which can smooth noise and preserve the edge information of the objects.

In Fig. 6b, the processed data is the hyperspectral remote sensing image of Pavia university scene. Obviously, the effect of PCA and EMP algorithms is very poor. They only recognize the ground objects with obvious spectral features, and others are regarded as background classes. The classification effect of RPNet and MGFEC algorithm is fairly good, which is close to the real situation on the ground.

For the Salinas Valley hyperspectral image data shown in Fig. 6c, the four methods can obtain most of the correct classification results. However, PCA and EMP algorithms have serious salt and pepper phenomenon, while RPNet and MGFEC algorithm have smooth and accurate classification effect, which is close to the ground truth.

In order to compare the experimental results more clearly, some experimental results are enlarged and displayed, such as the area marked by the red box in Fig. 6C,D. The details are shown in Figs. 7 and 8. Here Fig. 7 shows an enlargement of some of the experimental results in Fig. 6a. Figure 7a,b) show the experimental results of RPNet algorithm and MGFEC algorithm, respectively. In the same way, Fig. 8 is the enlargement effect of some results in Fig. 6c, and its physical meaning is the same as that in Fig. 7. It can be seen in Figs. 7 and 8 that the classification results obtained by MGFEC algorithm are more accurate, smoother and purer than those obtained by RPNet algorithm. Especially for the area objects with obvious shape boundary, the classification result is better, which can better reflect the real distribution of the region.

By comparing and analyzing the above experimental results, the following conclusions can be drawn. From the overall classification effect, MGFEC and RPNet methods have better classification effect, which is close to the distribution of ground truth, while PCA and EMP algorithms have poor classification effect. For the homogeneous block area object with large region, the classification effect of MGFEC method is better than RPNet method, and the quality of classification map is higher. Therefore, MGFEC method has better classification effect for hyperspectral images with obvious boundary ground objects.

### Quantitative analysis experiment

The analysis of the above experimental results and the comparison of algorithm performance are based on the visual effect of the image. The performance of the selected experimental algorithm is further analyzed and evaluated with quantitative indicators. The quantitative evaluation indexes include the overall accuracy (OA), the per-class accuracy (PA) and kappa coefficient.

The definition and calculation of these three evaluation parameters are based on a confusion matrix, which is also a square matrix. Assuming that the hyperspectral remote sensing image contains $c$ classes of objects, it is a square matrix of size $c \times c$. The element $u_{ii}$ on the diagonal of the matrix represents the number of correctly classified pixels for each class of ground object. The other elements of the matrix represent the number of pixels that are wrongly divided into another class.

1. Overall accuracy. The OA parameter refers to the sum of correctly classified pixels in hyperspectral images divided by the total number of samples. The calculation formula is as follows.
  13 $$ OA = \frac{{\sum\nolimits_{i = 1}^{c} {u_{ii} } }}{N} $$
  where $N$ represents the total number of image pixels, $u_{ii}$ represents the number of correctly classified pixels, and $c$ represents the number of ground target types. The higher the accuracy of classification is, the larger the OA value is, and its maximum value is one.
2. Per-class accuracy. The PA parameter of ground objects refers to the elements in each diagonal line of confusion matrix divided by the total number of elements in this line. Its calculation formula is as follows.
  14 $$ PA_{i} { = }\frac{{u_{ii} }}{{\sum\nolimits_{j = 1}^{c} {u_{ij} } }} $$
  where $u_{ij}$ is the number of pixels that are misclassified into class $i$ by other class $j$. If the PA value is larger, the classification accuracy of the class is more accurate, and the maximum value is one.
3. Kappa coefficient. Kappa coefficient is proposed by researchers to make up for the deficiency of OA as an evaluation of classification accuracy. It utilizes the information of the whole confusion matrix. The overall accuracy of classification only considers the number of pixels correctly classified in the diagonal direction, while kappa coefficient considers all kinds of missing and wrong pixels outside the diagonal, and its value range is [− 1,1], but it is usually greater than zero. It is a consistency test index, which is used to measure the accuracy of classification. The so-called consistency refers to whether the classified image is consistent with the reference image (the true classified image on the ground). The larger the value is, the higher the consistency is and the better the classification accuracy is. The calculation formula is as follows.
  15 $$ Kappa = \frac{{N(\sum\nolimits_{i = 1}^{c} {u_{ii} } ) - \sum\nolimits_{i = 1}^{c} {(\sum\nolimits_{j = 1}^{c} {u_{ij} } \sum\nolimits_{j = 1}^{c} {u_{ji} } )} }}{{(N^{2} - \sum\nolimits_{i = 1}^{c} {(\sum\nolimits_{j = 1}^{c} {u_{ij} } \sum\nolimits_{j = 1}^{c} {u_{ji} } )} )}} $$
  where $N$ is the sum of the number of all classes of ground objects in the image. $u_{ij}$, $u_{ii}$ and $u_{ji}$ are elements in the confusion matrix.

**Figure 9:**
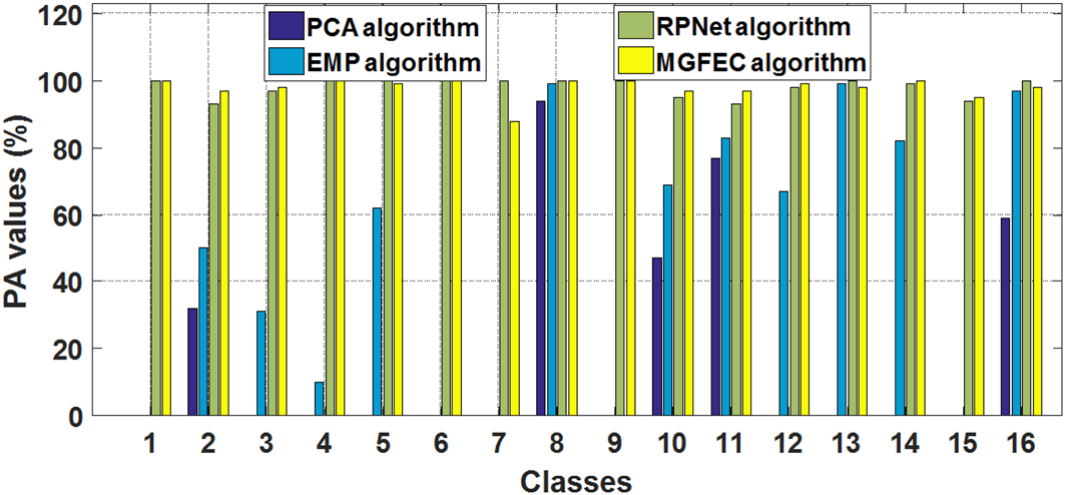
Comparison of PA values of different algorithms on the dataset shown in Fig. 2.

The results shown in Fig. 9 are the classification accuracy PA values of different algorithms on the Indian Pines dataset. In Fig. 9, the numbers "1–16" in the abscissa represent the class of the corresponding ground object on the dataset, the ordinate is the values of parameter PA and its unit is percentage. The images of Indian pines dataset contain 16 different classes of ground objects, so "1–16" in Fig. 9 corresponds to "1–16" in Table 2 in turn. Here different colors of the bar chart denote different methods, as shown in the legend. It can be seen in Fig. 9 that in the Indian pines dataset, the PA values obtained by MGFEC method and RPNet are significantly higher than those obtained by PCA and EMP. This shows that MGFEC and RPNet methods are better than the other two methods in classification effect. Moreover, for some classes of ground objects, such as class 2, class 10 and class 11 in Fig. 9 (class 2, class 10 and class 11 in Table 2), the PA value obtained by MGFEC method is significantly improved compared with RPNet method. This shows that MGFEC method is better than RPNet method in recognition and classification of these three classes of ground objects, because they have obvious boundaries in the image. This also fully shows that MGFEC method is suitable for classification of area objects with obvious and regular boundaries, and it can smooth the similar regions while preserving the image spatial edge information. This is the advantage of MGFEC method, because it uses the principle of guided filtering to enhance the edge and texture features, and uses the convolution kernel to extract abstract features, so it is conducive to improve the classification accuracy and quality of this kind of ground objects. For the datasets of Pavia University and Salinas Valley, the PA values reflect the same rule as the Indian pines dataset.

Table 5 shows the overall accuracy values and kappa coefficient values of each method classified under different datasets. It can be seen in Table 5 that for the three hyperspectral image datasets, the OA values obtained by PCA method only using spectral features are the smallest, and the kappa coefficient values are almost the smallest. This shows that the overall classification effect of PCA method is poor, and the consistency with the real type of ground is also poor. The OA values and kappa coefficients of EMP algorithm are higher than those of PCA algorithm, which indicates that the classification accuracy of EMP algorithm is improved. However, they are in the same order of magnitude and at the same level, and the difference between the effect and performance of them is not very big. For the remaining RPNet and MGFEC algorithms, their OA values and kappa coefficient values have been significantly improved, which are more than 90%, indicating that their classification performance and accuracy are good. The reason is that spatial features are introduced into the two methods, and their classification accuracy has been significantly improved. In contrast, the parameter value of MGFEC method is slightly higher than that of RPNet algorithm. In Indian Pines, Pavia University and Salinas Valley datasets, the OA value of MGFEC algorithm is higher than that of RPNet algorithm by 1.591%, 1.765% and 0.741%. This shows that MGFEC method is suitable for hyperspectral remote sensing image data classification, and the classification accuracy and effect are good, and it has good consistency with the ground true classification.

**Table 5:** OA value and Kappa coefficient value of different methods in different datasets.

Method	Parameter	Indian Pines dataset	Pavia University dataset	Salinas Valley dataset
PCA	OA	62.819%	79.760%	79.014%
	Kappa	0.137	0.006	0.398
EMP	OA	76.346%	83.745%	86.551%
	Kappa	0.281	0.061	0.331
RPNet	OA	96.464%	94.451%	95.451%
	Kappa	0.959	0.926	0.949
MGFEC	OA	**98.055%**	**96.216%**	**96.192%**
	Kappa	**0.977**	**0.950**	**0.957**

## Conclusions

Aiming at the problem that single scale feature cannot effectively express the differences between different objects and distinguish the boundaries of objects in hyperspectral remote sensing image classification, this paper utilizes the PCA theory to achieve the dimension reduction, abstract multi-scale features with guided filtering principle, and get the deep features through the convolution operation of random blocks. Finally, the SVM classifier is used to realize the classification of hyperspectral image. After the above processing steps, MGFEC algorithm not only obtains the structure information and deep-seated features of different scales of images, which is very conducive to the classification of hyperspectral images, but also improves the classification accuracy and quality of hyperspectral remote sensing images. A series of comparative experiments are carried out with actual hyperspectral remote sensing image data, and good experimental results are obtained. These show that the MGFEC algorithm proposed in this paper can not only effectively extract multi-scale features, but also better combine spatial and spectral information, enhance the discrimination ability between classes, and greatly improve the image classification accuracy. Therefore, it is a feasible method, and has some practical value.
